# Nose-to-brain delivery of temozolomide-loaded PLGA nanoparticles functionalized with anti-EPHA3 for glioblastoma targeting

**DOI:** 10.1080/10717544.2018.1494226

**Published:** 2018-09-03

**Authors:** Liuxiang Chu, Aiping Wang, Ling Ni, Xiuju Yan, Yina Song, Mingyu Zhao, Kaoxiang Sun, Hongjie Mu, Sha Liu, Zimei Wu, Chunyan Zhang

**Affiliations:** a School of Pharmacy, Collaborative Innovation Center of Advanced Drug Delivery System and Biotech Drugs in Universities of Shandong, Yantai University, Yantai, China;; b State Key Laboratory of Long-Acting and Targeting Drug Delivery System, Shandong Luye Pharmaceutical Co., Ltd, Yantai, China

**Keywords:** Glioblastoma, EPHA3 antibody, temozolomide butyl ester, nose-to-brain delivery, nanoparticles

## Abstract

Glioblastoma is the most common malignant brain tumor. Efficient delivery of drugs targeting glioblastomas remains a challenge. Ephrin type-A receptor 3 (EPHA3) tyrosine kinase antibody-modified polylactide-co-glycolide (PLGA) nanoparticles (NPs) were developed to target glioblastoma via nose-to-brain delivery. Anti-EPHA3-modified, TBE-loaded NPs were prepared using an emulsion-solvent evaporation method, showed a sustained *in vitro* release profile up to 48 h and a mean particle size of 145.9 ± 8.7 nm. The cellular uptake of anti-EPHA3-modified NPs by C6 cells was significantly enhanced compared to that of nontargeting NPs (*p* < .01). *In vivo* imaging and distribution studies on the glioma-bearing rats showed that anti-EPHA3-modified NPs exhibited high fluorescence intensity in the brain and effectively accumulated to glioma tissues, indicating the targeting effect of anti-EPHA3. Glioma-bearing rats treated with anti-EPHA3-modified NPs resulted in significantly higher tumor cell apoptosis (*p* < .01) than that observed with other formulations and prolonged the median survival time of glioma-bearing rats to 26 days, which was 1.37-fold longer than that of PLGA NPs. The above results indicated that anti-EPHA3-modified NPs may potentially serve as a nose-to-brain drug carrier for the treatment of glioblastoma.

## Introduction

Glioblastoma multiforme (GBM) is the most common primary central nervous system tumor with an incidence of 5-8 per 100,000 population. This tumor is highly aggressive, and the 5-year survival rate is lower than 5% (Gao et al., 2014). Although significant progress has been made in surgery, neuroimaging, radiotherapy, and chemotherapy, the prognosis for patients with GBM is still poor, and the median survival is less than 1 year (Attenello et al., 2008; Combs et al., 2008). Even the most active chemotherapy treatment available can only slightly improve the total survival rate. Temozolomide (TMZ), a DNA-alkylating agent, has been found to be the most effective antineoplastic agent for treating GBM by orally or intravenously (Sharma et al., 2017) and has been approved by the U.S. Food and Drug Administration for clinical use (Yung et al., 2000). Unfortunately, owing to its short serum half-life and dose-limiting side effects, systemic delivery of TMZ as a supplement to radiation therapy only results in moderate benefits (Baker et al., 1999; Chakravarti et al., 2006). Furthermore, prolonged systemic administration of TMZ is associated with some toxic side effects such as hematological toxicity, acute cardiomyopathy, oral ulceration, and myelosuppression (Trinh et al., 2009). To avoid these problems, at present, microsphere formulations, topical implants and nanoformulations of TMZ have been widely studied for the study and treatment of GBM (Zhang & Gao, 2007; Song et al., 2016). Among them, the nanoparticle (NP)-based drug delivery systems for research and treatment of GBM seems to be more favored by the majority of researchers (Ling et al., 2012; Ramalho et al., 2018). In these systems, polylactide-co-glycolide (PLGA) shows great promise for applications in drug delivery because of its biodegradability, biocompatibility, and versatility. However, the poor solubility of TMZ in aqueous and organic media has led to significant difficulties in its encapsulation into PLGA-NPs (P-NPs) (Lee & Ooi, 2016). Therefore, Wang (2009) have introduced a carbon chain of 4–10 carbons to the TMZ molecule, and successfully synthesized TMZ esters, which were demonstrated to show activity comparable with that of TMZ. However, to date, no studies have reported the use of TMZ esters in nanoformulations.

At present, most researches for the treatment of GBM are aimed at penetrating the blood–brain barrier (BBB) and delivering drugs into the brain to the site of the lesion (Gao, 2016). Previous studies have designed the dual-targeted nano delivery systems (Ruan et al., 2017) and targeting gold nanocarrier delivery system (Ruan et al., 2015) to penetrate the BBB and achieved good therapeutic effect, providing new therapeutic strategies for GBM. This article aims to design a drug delivery system for the treatment of GBM via the nose-to-brain delivery. It is undeniable that compared with other routes of administration, a nose-to-brain targeting system could provide many advantages for the treatment of GBM. The neural connection between the nasal mucosa and the brain provides a unique approach to nonoffensive delivery of drugs (Khan et al., 2017). The intranasal route allows direct access of a drug to the brain through olfactory and trigeminal nerve pathways, and systemic side effects are avoided by bypassing the BBB. In addition, it avoids first-pass metabolism and prevents enzymatic/chemical degradation of the drug. It works promptly and reduces the dose and frequency of its administration, thereby enhances patient compliance. However, limitations of nasal mucociliary clearance, which seriously affects the sustainable drug absorption, need to be considered during administration (Djupesland, 2013).

Mucosal adhesion polymers can be used to increase the nasal retention time (Djupesland, 2013). Chitosan is commonly used as a nasal adhesive, but it does not dissolve or adhere under neutral pH conditions (Gartziandia et al., 2015). However, *N*-trimethylated chitosan (TMC), prepared via reductive methylation of chitosan, shows good adhesion and solubility (Hagenaars et al., 2010). Researchers have used TMC to enhance the adhesion to and overcome the clearance from the nasal cavity (du Plessis et al., 2010).

To facilitate the drug delivery to GBM, some specific ligands are often used to target receptors overexpressed in GBM (Hwang et al., 2011; Fan et al., 2018). Ephrin type-A receptor 3 (EPHA3), a membrane-associated receptor, which is particularly overexpressed in stroma and vasculature in gliomas but not in normal tissues, can be used as a functional target for the treatment of GBM (Janes et al., 2014). Anti-EPHA3, a first-rate recombinant, nonfucosylated IgG1κ (human f-allotype) monoclonal antibody, can target the EPHA3 receptor tyrosine kinase (Day et al., 2013; Swords et al., 2016). Moreover, anti-EPHA3 has been applied in preclinical models and shown significant efficacy and low toxicity (Vail et al., 2014). Currently, anti-EPHA3 (KB004) has entered a phase I clinical trial. These findings indicate that anti-EPHA3 is a suitable ligand to enhance the nose-to-brain delivery for targeting GBM during nasal administration.

Herein, we developed a nose-to-brain targeted nanodelivery system using anti-EPHA3-modified P-NPs for the treatment of GBM. The anti-EPHA3 modification was aimed at targeting GBM, while TMC coating was intended to increase the adhesion to the nasal mucosa.

## Materials and methods

### Materials and animals

TMZ was purchased from Wuhan Fuxin Chemical Co., Ltd. (Wuhan, China). PLGA 5050 2 A (lactide/glycolide ratio: 50/50; molecular weight: 18,000) was provided by Shandong Luye Pharmaceutical Co., Ltd. (Yantai, China). The EPHA3 antibody, dimethyl sulfoxide (DMSO), and 3-(4,5-dimethylthiazol-2-yl)-2,5-diphenyltetrazolium bromide (MTT) were obtained from Sigma − Aldrich, Inc. (St. Louis, MO). Nile red, coumarin-6, Hoechst 33342, and 1,-1-dioctadecyl-3,-3,-3′,-3′-tetramethylindotricarbocyanine (DiR) iodide were purchased from Aladdin Industrial Corp. (Shanghai, China). The bicinchoninic acid (BCA) protein quantification kit and the terminal deoxyribonucleotide transferase-mediated dUTP nick-end labeling (TUNEL) apoptosis detection kit were purchased from Beyotime Biotechnology (Shanghai, China). The optimal cutting temperature (OCT) compound was purchased from Sakura Finetek USA, Inc. (Torrance, CA). The human EPHA3 enzyme-linked immunosorbent assay (ELISA) kit was purchased from Elabscience Biotechnology Co., Ltd. (Wuhan, China). All reagents were of analytical grade.

Dulbecco’s modified Eagle’s medium (DMEM, Invitrogen, Carlsbad, CA) and heat-inactivated fetal bovine serum were provided by Invitrogen. The 16HBE and C6 cell lines were provided by the American Type Culture Collection (Zhongyuan, Ltd., Beijing, China).

Adult Sprague-Dawley male rats were purchased from Vital River Laboratory Animal Technology Co., Ltd. (Beijing, China). All animal experiments were conducted in accordance with the guidelines of the Ethical Committee on Animal Experimentation of Yantai University (Yantai, China) and in compliance with the EC Directive 2010/63/EU and the National Institutes of Health guidelines on animal welfare.

### Synthesis and characterization of TBE

Briefly, TMZ was weighed and dissolved in concentrated sulfuric acid with stirring, and then a saturated aqueous solution of sodium nitrite was added dropwise at 15 °C, with stirring continued until the end of the reaction. The collected product was washed and dried and then added to an appropriate amount of anhydrous dimethylformamide. Subsequently, then n-butanol, dicyclohexylcarbodiimide, and 4-dimethylaminopyridine were added and reacted at room temperature. The whole process was monitored by high-performance liqid chromatography (HPLC), and the final product, temozolomide butyl ester (TBE), was verified using a ^1^H-nuclear magnetic resonance (^1^H-NMR) spectrometer.

#### Preparation of NPs

An emulsion-solvent evaporation method was used to prepare NPs. Briefly, PLGA and TBE were dissolved in acetone and dichloromethane to form an oil phase. The oil phase was added dropwise to 1% (w/v) polyvinyl alcohol in deionized water, with sonication in an ice bath, and the mixture was stirred under reduced pressure to remove the organic solvents. NPs were collected by centrifugation and washed with deionized water to remove free TBE. A single-factor study was performed, with the particle size and drug loading as evaluation indexes, to examine the effects of the oil-phase composition, oil/water ratio, polymer concentration, and theoretical drug loading. Nile red-, coumarin-6- and DiR-loaded NPs were prepared in the same way, and used to evaluate the uptake and distribution of NPs in the brain.

TMC and maleimide (Mal)-TMC were synthesized and verified by ^1^H-NMR according to the report of Meng et al. (2018). TMC was applied to coat P-NPs for the preparation of TMC/PLGA-NPs (T/P-NPs), and anti-EPHA3 was conjugated with Mal-TMC-coated PLGA-NPs to prepare anti-EPHA3-TMC/PLGA-NPs (anti-EPHA3-T/P-NPs). Anti-EPHA3 was thiolated by reacting with a 20:1 M excess of Traut’s reagent for 1 h according to Huwyler’s method (Huwyler et al., 1996). The product was purified using a Sephadex G25 column, and the purified thiolated antibody was coupled to Mal-T/P-NPs at an anti-EPHA3:Mal molar ratio of 1:5 for 8 h at room temperature. Anti-EPHA3-T/P-NPs were purified to remove unconjugated antibody using gel filtration. The antibody conjugation efficiency was assessed using the BCA protein quantification kit.

#### Characterization of NPs

The average diameter, polydispersity index, and zeta potential of NPs were measured using a dynamic light scattering particle size analyzer (Deisa TM Nano C; Beckman Coulter, Brea, CA). Drug loading of NPs was measured by an ultraviolet (UV) spectrophotometric method. Samples were placed in an ultrafiltration device (100-kDa molecular weight cutoff; Sartorius, Göttingen, Germany) and centrifuged at 3,600 rpm for 10 min at 4 °C to separate the free drug. The total drug content was analyzed using the same volume of a sample dissolved in acetonitrile. The mobile phase consisted of methanol and 0.5% acetic acid (3:1, v/v). The flow rate was maintained at 1 mL/min, and the UV detection was performed at 327 nm. The concentrations of TBE and coumarin-6 were measured using an HPLC system (LC-20A VP; Shimadzu, Kyoto, Japan), while those of Nile red and DiR were measured by UV spectrophotometry. The drug-loading capacity of NPs was calculated using the following equation:

(1) $$\text{Drug loading }\left(\%\right)=\frac{\text{Total}\;\text{amount}\;\text{of}\;\text{drug}-\text{Amount}\;\text{of}\;\text{free}\;\text{drug}}{\text{Weight of NPs}}\times 100\%\;$$

### *In vitro* TBE release study

NPs were dialyzed in 15 mL of 0.1 M phosphate-buffered saline (PBS), pH 7.4, for 96 h at 37 °C on a rocker. At predefined time intervals, 1 mL of the PBS was withdrawn from outside the dialysis bag, and replaced with an equivalent volume of the release medium. Samples were collected at various times (0–96 h) and analyzed for TBE using HPLC as described above. A solution of TBE in PBS was used as the control.

### Analysis of EPHA3 expression

To confirm specific expression of EPHA3 on GBM, human bronchial epithelial (16HBE) cells, C6 cells, and glioma tissues were used to detect EPHA3 by ELISA. The double antibody sandwich ELISA method was used according to the kit manual, and the anti-human EPHA3 antibody was coated on the enzyme-labeling board. The cells and tissues were collected and homogenized, and total protein concentrations were measured in the supernatants by the BCA assay. The concentration of EPHA3 was measured by ELISA according to the manufacturer’s instructions using the same concentrations of total protein in the supernatant. The following formula was used to calculate the percentage of EPHA3 expression:

(2) $$\text{EPHA}3\;\left(\%\right)=\frac{c_{\text{EPHA}3}}{c_{\text{total protein}}}\times 100\%\;$$

### *In vitro* cell study

#### *In vitro* cytotoxicity assay

16HBE and C6 cells were maintained in a growth medium composed of Dulbecco’s modified Eagle’s medium (Invitrogen) supplemented with 10% fetal bovine serum (Invitrogen), 100 IU/mL penicillin, and 100 mg/mL streptomycin sulfate. The cells were grown and maintained in a humidified atmosphere containing 5% CO_2_ at 37 °C.

The cytotoxicity of TBE-loaded NPs for C6 and 16HBE cells was evaluated using an MTT assay. Briefly, cells were seeded in 96-well plates at a density of 5 × 10^3^ cells/well and incubated for 24 h under 5% CO_2_ at 37 °C. Then, 16HBE cells were treated with TBE-loaded or unloaded NPs for 6 h, while C6 cells were treated with the above formulations for 48 h. After incubation for predefined times, an MTT solution (20 μL) was added to each well, followed by incubation for 4 h. Then, the media were removed, and 200 μL of DMSO was added. Absorbance was measured using a microplate reader (SpectraMax M2, Molecular Devices, San Jose, CA) at a wavelength of 570 nm after gentle shaking for 10 min. Cell viability was determined by comparing the absorbance of NP-treated cells with that of control samples.

#### Cellular uptake study

To explore the cellular uptake of NPs, C6 cells were incubated with Nile red-loaded NPs and qualitatively analyzed by fluorescence microscopy (Eclipse E400; Nikon Corporation, Tokyo, Japan), while quantitative analysis of coumarin-6-loaded NPs was performed using flow cytometry (BD Accuri™ C6 Plus; BD Biosciences, Franklin Lakes, NJ).

For qualitative analysis, C6 cells were seeded into 24-well plates (1 × 10^5^ cells in 1 mL of the medium per well) and incubated at 37 °C under 5% CO_2_. After 24 h, the cells were incubated with the same concentrations (1 µg/mL) of Nile red-loaded P-NPs, T/P-NPs, or anti-EPHA3-T/P-NPs for 0.5, 1, and 2 h. After the incubation, the cells were washed three times with PBS and fixed with 4% paraformaldehyde at room temperature for 10 min. Image analysis was performed using fluorescence microscopy.

For quantitative analysis, C6 cells were seeded into 6-well plates (4 × 10^5^ cells in 2 mL of the medium per well) and incubated at 37 °C under 5% CO_2_. After 24 h, the cells were incubated with the same concentrations (4 ng/mL) of coumarin-6-loaded P-NPs, T/P-NPs, or anti-EPHA3-T/P-NPs for 0.25, 0.5, 1, and 2 h. The cells were then trypsinized, collected by centrifugation, and washed three times with PBS. Finally, 1 × 10^4^ cells were analyzed by flow cytometry to determine the uptake of coumarin-6.

### *In vivo* study

#### Model of glioma-bearing rats

Male Sprague-Dawley rats (weighing 180–220 g) were maintained at 22 °C on a 12-h light/dark cycle in polyethylene cages with ad libitum access to food and water. GBM development was induced by intracranial implantation of C6 cells. Briefly, rats were anesthetized by intraperitoneal (i.p.) injection of 10% chloral hydrate (0.4 mL/kg), and then C6 cells (1 × 10^6^ cells in 6 μL of DMEM) were injected stereotactically into the right caudate nucleus (3 mm lateral to the bregma and 5 mm deep from the dura) using a microsyringe (Hua et al., 2018). The wounds were losed with sutures and sterilized with iodophor. Penicillin was injected intramuscularly, and the rats were carefully monitored until recovery from anesthesia.

#### *In vivo* imaging and brain distribution of NPs

The intracranial glioma-bearing rats were randomly divided into four groups, of which three groups were administered DiR-loaded P-NPs, T/P-NPs, and anti-EPHA3-T/P-NPs, respectively, through the nasal mucosa, and the last group was injected intravenously via the tail vein with DiR-loaded anti-EPHA3-T/P-NPs (0.5 mg/kg). Real-time imaging was performed at predetermined time points (1, 2, 4, and 8 h) using an *in vivo* imaging system. Subsequently, some rats were sacrificed, and organs, including the brain, heart, liver, spleen, lungs, and kidneys, were harvested for imaging.

For qualitative analysis of the brain biodistribution of NPs, glioma-bearing rats were randomly divided into three groups 14 days after C6 cell implantation and administered intranasally with coumarin-6-loaded P-NPs, T/P-NPs, and anti-EPHA3-T/P-NPs (0.5 mg/kg), respectively. Four hours after the administration, the rats were anesthetized by i.p. injection of 10% chloral hydrate, and then heart perfusion was performed with saline and 4% paraformaldehyde. The rat brains were harvested, fixed in 4% paraformaldehyde for 24 h, and dehydrated in 15% and 30% sucrose solutions. The brains were then embedded in the Tissue-Tek OCT compound at −80 °C and cut into 10 μm sections. The sections were stained with Hoechst 33342 for 5 min and washed three times with PBS. Finally, the sections were imaged and analyzed using a fluorescence microscope.

#### *In vivo* anti-glioma activity

To evaluate the *in vivo* anti-glioma activity of various formulations, intracranial glioma-bearing rats were divided into five groups which were administered intranasally with saline, free TBE, P-TBE-NPs, T/P-TBE-NPs, and anti-EPHA3-T/P-TBE-NPs, respectively, at a TBE dose of 5 mg/kg. Administrations were performed at 6, 7, 8, 9, and 10 days after C6 cell implantation. On the 15th day, one rat from each group was sacrificed, and the brain was isolated to prepare paraffin sections (10 μm) (Kumari et al., 2017). A TUNEL apoptosis detection kit (color development method) was used to detect apoptotic glioma cells, which were counted using the Image-Pro Plus 5 software (Media Cybernetics, Inc., Rockville, MD). The other rats (10 per group) were used to calculate survival times.

### Statistical analysis

Data are expressed as the mean ± SD. Statistical significance was analyzed using one-way analysis of variance or Student's *t*-test, with *p* < .05 indicating statistical significance and *p* < .01 considered highly statistically significant.

## Results and discussion

### Characterization of TBE

TBE synthesis was verified by ^1^H-NMR. The peaks of TMZ at 3.88 ppm (-CH_3_), 7.6-7.7 ppm (-CONH_2_), and 8.77 ppm (-CH) were confirmed. Chemical shifts of TBE at 4.45 ppm (-O-CH_2_-C-), 1.79 ppm (-C-CH_2_-C-), and 1.46 ppm (-C-C-CH_3_-), indicating the successful synthesis of TBE. The purity of TBE reached 98% based on liquid phase detection, also indicating that the synthesis of TBE was successful.

### Characterization of NPs

We selected conditions to prepare TBE-loaded NPs, as described in the Materials and Methods section, based on the results of a single-factor investigation, presented in Table 1. The particle sizes, zeta potentials, and polydispersity indexes of various NPs are provided in Table 2. It was found that the particle sizes of TBE-loaded NPs ranged from 120 to 160 nm, which met the requirement for the diameter of NPs to be used for nasal administration, although the mean diameters of T/P-NPs and anti-EPHA3-T/P-NPs were greater than that of P-NPs, owing to the modification with TMC and binding of anti-EPHA3. As expected, TMC-coated NPs were positively charged, while P-NPs had a negative zeta potential (Sheng et al., 2015). The polydispersity indexes of less than 0.2 indicated that the particle size distribution of NPs was uniform. The drug-loading capacity of the anti-EPHA3-T/P-NPs prepared by the emulsion-solvent evaporation method was 3.02 ± 0.68%. The conjugation efficiency of anti-EPHA3 was 8.1 ± 1.5%.

**Table 1.** Single factor study of TBE-loaded P-NPs obtained under different conditions.

Sample	Different conditions	Diameter (nm)		Drug loading (%)
Oil-phase composition (1mL)				
1	dichloromethane	200.5		2.85
2	dichloromethane:acetone = 1:1	147.8		1.65
3	dichloromethane:acetone = 4:1	121.6		3.26
Oil/water ratio				
4	1:3	131.1		2.92
5	1:5	142.2		2.39
6	1:10	190.3		0.89
Polymer concentration (mg/mL)				
7	5	144.5		1.68
8	10	145.7		2.34
9	20	150.8		1.17
Theoretical drug loading (%)				
10	5	144.9		0.78
11	10	141.8		2.49
12	15	144.2		2.10

**Table 2.** Characterization of TBE-loaded NPs.

Formulation	Diameter (nm)	Polydispersity Index	Zeta potential (mV)	Drug loading (%)
P-NPs	125.1 ± 3.5	0.084 ± 0.020	−21.05 ± 1.5	3.54 ± 0.51
T/P-NPs	130.3 ± 4.5	0.102 ± 0.042	+25.18 ± 2.3	3.23 ± 0.43
anti-EPHA3-T/P-NPs	145.9 ± 8.7	0.121 ± 0.035	+23.08 ± 2.5	3.02 ± 0.68

NPs: nanoparticles.

### *In vitro* TBE release study

**Figure 1.**
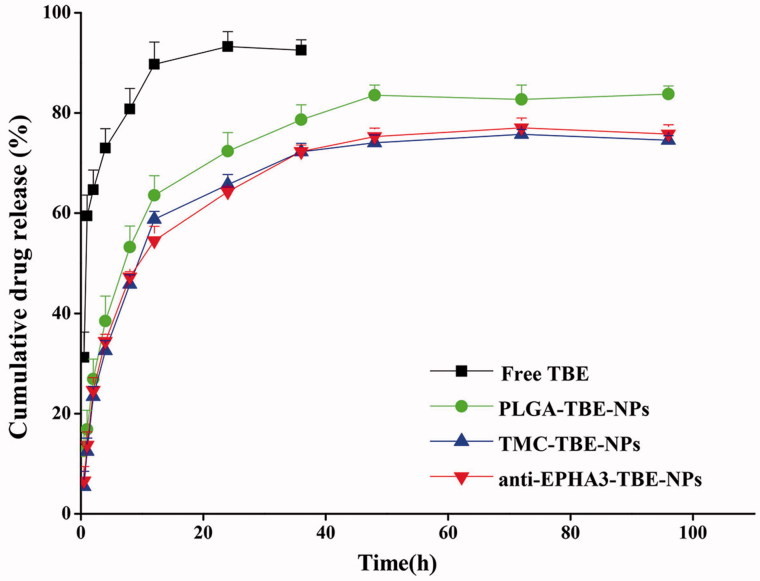
*In vitro* TBE release profiles of free TBE, P-TBE-NPs, T/P-TBE-NPs, and anti-EPHA3-T/P-TBE-NPs.

The drug release profiles of the samples are exhibited in Figure 1. The free TBE was released rapidly (within 4 h) from the dialysis bag, while TBE-loaded P-NPs, T/P-NPs, and anti-EPHA3-T/P-NPs achieved a sustained release of TBE for up to 48 h.

### EPHA3 expression

As mentioned in the Introduction section, EPHA3 is only expressed in glioma cells, especially in tumor stroma and vasculature, but not in normal cells (Janes et al., 2014). In our study, we selected C6 (EPHA3^+^) and 16HBE (EPHA3^—^) as model cells and first confirmed the expression of EPHA3. The expression levels of EPHA3 in glioma tissues and C6 cells were 4.06 ± 0.2% and 2.49 ± 0.15%, respectively, while almost no expression was detected in 16HBE cells.

### *In vitro* cytotoxicity

To evaluate the safety of anti-EPHA3-T/P-NPs for nasal administration, the 16HBE cell line was used as a model of nasal mucosa cells (Bi et al., 2016). The duration of the cytotoxicity experiment was set to 6 h because of the nasal scavenging capacity. The results show that no significant differences were observed among TBE-loaded or unloaded P-NPs, T/P-NPs, and anti-EPHA3-T/P-NPs in their effects on cell viability, indicating that it is safe and feasible to deliver TBE into the brain through the nasal mucosa using anti-EPHA3-T/P-NPs as a carrier.

To evaluate the cytotoxicity of TBE-loaded NPs for tumor cells, C6 cells were incubated with different concentrations of various TBE-loaded NPs, and cell viability was inhibited by NPs in a concentration-dependent manner. At each preset concentration, anti-EPHA3-T/P-TBE-NPs showed the strongest inhibition of C6 cell growth, followed by T/P-TBE-NPs, suggesting that the anti-EPHA3-modified NPs had the ability to target C6 cells, which was likely due to anti-EPHA3 binding to the receptor, thus increasing the cellular uptake of NPs. For instance, the cell viability of anti-EPHA3-T/P-TBE-NPs was 25.76% at 60 µg/mL of TBE, while the cell viability in T/P-TBE-NPs and P-TBE-NPs were 42.40% and 43.15%, respectively. Further, the vehicle-associated cytotoxicity was found to be negligible in the concentration ranges of PLGA of 0.23-2.35 mg/mL. In addition, the effects of T/P-NPs on cell viability were stronger than those of the uncoated group, which might be attributed to the fact that positively charged T/P-NPs were more prone to attaching to negatively charged cells (Meng et al., 2018).

### Cellular uptake study

**Figure 2.**
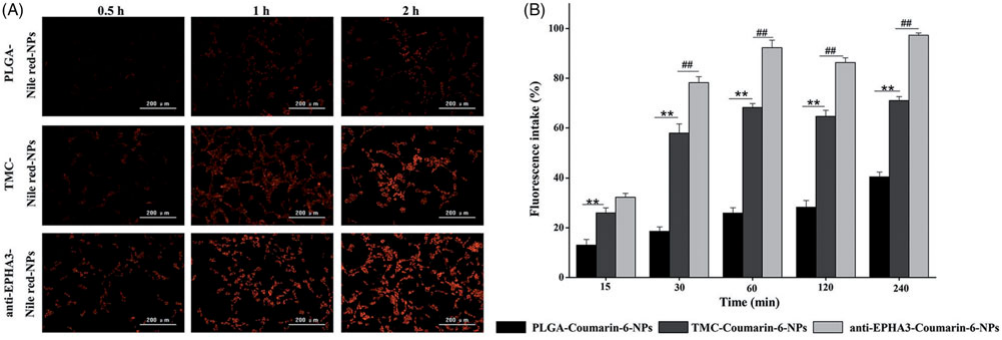
(A) Fluorescence microscopy images of C6 cells incubated with Nile red-loaded NPs. (B) Mean percentages of coumarin-6 NP uptake by C6 cells, as determined by flow cytometry. Values represent the mean ± SD (*n* = 3). **p < .01 versus P-NPs; ##*p* < .01 versus T/P-NPs. NPs: nanoparticles.

Fluorescence microscopy was used to qualitatively evaluate the uptake of NPs by C6 cells, as shown in Figure 2(A). The cellular uptake of NPs occurred in a time-dependent manner. C6 cells treated with anti-EPHA3-modified NPs emitted stronger fluorescence than those treated with unmodified NPs did, and the fluorescence intensity in the T/P-NP uptake group was higher than that in the P-NP uptake group. Similar to the results of quantitative analysis by flow cytometry (Figure 2B), the uptake of T/P-NPs was significantly higher than that of P-NPs at each tested time point (*p* < .01), indicating that TMC adhesion could improve the cellular uptake, as in a previous study (Drin et al., 2003). Furthermore, there were significant differences between anti-EPHA3-T/P-NPs and T/P-NPs (*p* < .01) from 0.5 to 4 h, indicating that the modification with anti-EPHA3 could greatly increase the cellular uptake of NPs.

### *In vivo* imaging and brain distribution of NPs

**Figure 3.**
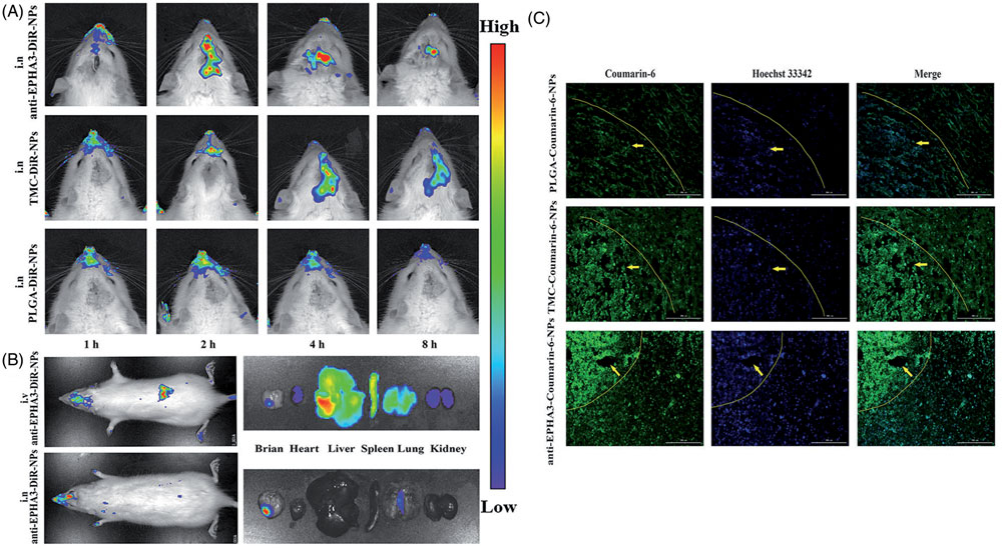
*In vivo* and brain distribution of DiR- and coumarin-6-loaded NPs in glioma-bearing rats. (A) *In vivo* fluorescence imaging at predetermined time points after intranasal administration of DiR-loaded NPs. (B) *In vivo* and excised tissues imaging of anti-EPHA3-modified DiR-loaded NPs at 4 h after intranasal and intravenous administration. (C) Fluorescence microscopy images of the brain, acquired 4 h after intranasal administration of coumarin-6-loaded NPs to glioma-bearing rats. Green: coumarin-6; blue: Hoechst 33342 (nuclei); yellow arrows point to the tumor site. DiR, 1,-1-dioctadecyl-3,-3,-3′,-3′-tetramethylindotricarbocyanine; NPs: nanoparticles.

To evaluate the glioma-targeting efficacy of anti-EPHA3-T/P-NPs *in vivo*, glioma-bearing rats were administered DiR-loaded P-NPs, T/P-NPs, and anti-EPHA3-T/P-NPs, and real-time *in vivo* imaging was performed. The distribution of NP fluorescence in the rats at 1, 2, 4, and 8 h after nasal administration is shown in Figure 3(A). The fluorescence signal of anti-EPHA3-T/P-NPs in the brain was higher than those of T/P-NPs and P-NPs, indicating that the anti-EPHA3-modified NPs could deliver more drug into the brain via intranasal administration. To prove that nasal administration can increase the drug delivery into the brain and reduce systemic distribution, the distribution of fluorescence in the rats was compared for intravenous and intranasal administration. The distribution of fluorescence in the rats and excised tissues at 4 h post-nasal and post-intravenous administration of DiR-loaded anti-EPHA3-T/P-NPs is shown in Figure 3(B). The NP fluorescence was accumulated in the brain, especially at the tumor site, in the rats administered intranasally, with a small amount of fluorescence detected in the lungs because of inhalation from the nasal cavity. Meanwhile, after intravenous administration, the fluorescence was distributed throughout the body, mostly in the liver, indicating that nasal administration results in the delivery of more drug into the brain and reduces peripheral distribution. Overall, the above results indicated that anti-EPHA3-modified NPs enhance the drug delivery for glioma targeting via the nose-to-brain route. Therefore, this delivery mode was used to evaluate the anti-EPHA3-modified NPs for targeted therapy of GBM.

To further observe the distribution of NPs in the brain, cryosections of glioma-bearing rats were prepared at 4 h after nasal administration of NPs. In Figure 3(C), the nuclei are stained with Hoechst 33342 (blue), and NPs are green. Slight and uniform distribution of P-NPs was observed in the section, while clearly higher fluorescence intensity was found in the T/P-NP section, suggesting that TMC adhesion could promote NP delivery into the brain. In particular, anti-EPHA3-T/P-NPs exhibited a higher fluorescence signal at the glioma site compared with that exhibited by T/P-NPs and P-NPs, indicating that anti-EPHA3-modified NPs were more likely to enter the brain and accumulate within the tumor.

### *In vivo* anti-glioma activity

**Figure 4.**
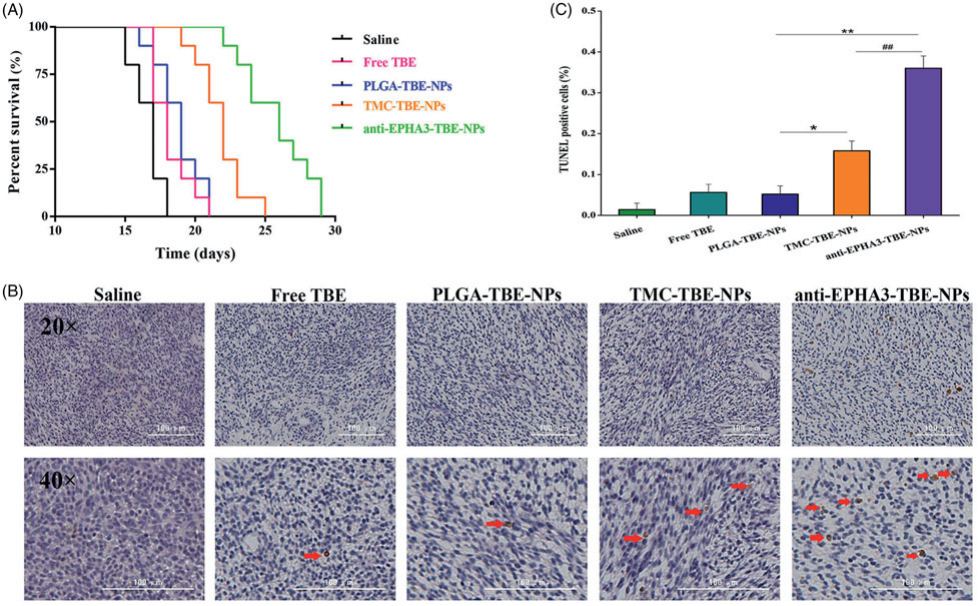
Effects of treatments with TBE-loaded NPs on glioma-bearing rats. (A) Survival curves of glioma-bearing rats after different treatments (*n* = 10 each). (B) Apoptosis of glioma cells in glioma-bearing rats treated with saline, free TBE, P-TBE-NPs, T/P-TBE-NPs, and anti-EPHA3-T/P-TBE-NPs. Brown: apoptotic tumor cells; blue: tumor cells; red arrows point to apoptotic cells. (C) Quantification of apoptosis in rats treated with different formulations. Values represent the mean ± SD (*n* = 3). *p < .05 and **p < .01 versus P-TBE-NPs; #*p* < .05 versus T/P-TBE-NPs. NPs: nanoparticles; TBE: temozolomide butyl ester.

Glioma-bearing rats were used to evaluate the anti-glioma effects of TBE-loaded NPs, with or without targeting ligands. As shown in Figure 4(A), the median survival times were 17, 18, and 19 days in the saline, free TBE, and P-TBE-NP groups with no statistical differences. Meanwhile, modification of NPs with anti-EPHA3 resulted in a better anti-glioma effect, with the 1.52-, 1.44-, 1.37- and 1.18-fold longer median survival time than those in the saline, free TBE, P-TBE-NPs, and T/P-TBE-NPs groups, respectively, indicating that the anti-EPHA3 modification could improve the anti-glioma activity of TBE. The same conclusion could also be made based on TUNEL staining of the brain tumor (Figure 4B). These images demonstrate that the apoptosis of glioma cells significantly increased in the glioma-bearing rats treated with anti-EPHA3-T/P-TBE-NPs compared with that in the rats treated with the other formulations. The results of Figure 4(C), show that the percentage of apoptotic glioma cells in the anti-EPHA3-T/P-TBE-NP group was significantly higher than that in the T/P-TBE-NP group (*p* < .05) and in the P-TBE-NP group (*p* < .01). These results confirmed that the enhanced anti-glioma effects were attributed to the anti-EPHA3 modification.

## Conclusions

In summary, anti-EPHA3-modified TBE-loaded P-NPs, TMC coated were successfully prepared. An *in vitro* cell viability assay using the 16HBE cell line supported safety of the developed NPs for intranasal administration. The results of a C6 cell cytotoxicity assay and subsequent experiments on specific cellular uptake of NPs demonstrated that the anti-EPHA3 modification could enhance GBM targeting. The fluorescence distribution and anti-glioma efficacy in glioma-bearing rats confirmed the utility of this formulation in the treatment of GBM. These results indicate that anti-EPHA3-T/P-NPs can potentially be used as a drug delivery system for nose-to-brain targeted therapy of GBM.
